# 770 Rehabilitation Evaluation and Treatment for the Management of Skin Graft Complication of the Genitalia

**DOI:** 10.1093/jbcr/irae036.311

**Published:** 2024-04-17

**Authors:** Chloe Tremblay, Stephanie Jean, Genevieve Schneider, Bernadette Nedelec, Zoë Edger-Lacoursière

**Affiliations:** Villa Medica Rehabilitation Hospital, Montréal, QC; Universite de Montreal, Montreal, QC; Villa Medica Rehab Center, Montréal, QC; McGill University, Montreal, QC; Villa Medica Rehabilitation Hospital and McGill University, Montréal, QC; Villa Medica Rehabilitation Hospital, Montréal, QC; Universite de Montreal, Montreal, QC; Villa Medica Rehab Center, Montréal, QC; McGill University, Montreal, QC; Villa Medica Rehabilitation Hospital and McGill University, Montréal, QC; Villa Medica Rehabilitation Hospital, Montréal, QC; Universite de Montreal, Montreal, QC; Villa Medica Rehab Center, Montréal, QC; McGill University, Montreal, QC; Villa Medica Rehabilitation Hospital and McGill University, Montréal, QC; Villa Medica Rehabilitation Hospital, Montréal, QC; Universite de Montreal, Montreal, QC; Villa Medica Rehab Center, Montréal, QC; McGill University, Montreal, QC; Villa Medica Rehabilitation Hospital and McGill University, Montréal, QC; Villa Medica Rehabilitation Hospital, Montréal, QC; Universite de Montreal, Montreal, QC; Villa Medica Rehab Center, Montréal, QC; McGill University, Montreal, QC; Villa Medica Rehabilitation Hospital and McGill University, Montréal, QC

## Abstract

**Introduction:**

Skin graft complications can include pain, contractures, hypertrophic scars, sensitivity issues, recurrent wounds and infections. Genitalia, perineum and/or buttocks graft complications have a direct impact on walking, sitting, voiding, bowel elimination, sexual function, intimacy, thereby reducing quality of life. Perineal and pelvic rehabilitation is used for many pelvic floor disorders, however, has not been described after burn injury or necrotizing fasciitis. Thus, this case report describes, a new rehabilitation evaluation template following perineal grafting and treatment outcomes reported after necrotizing fasciitis.

**Methods:**

Assessments included Patient and Observer Scar Assessment Scale (POSAS), penile deviation measure, sensitivity using monofilaments, Burn Survivor Fear Avoidance Questionnaire (BSFAQ), sexual function and satisfaction. Evaluation and treatment were conducted by a certified pelvic floor physiotherapist (PT) and occupational therapist (OT). The initial evaluation occurred 4 months post surgery with interventions occurring every 3-4 weeks thereafter.

**Results:**

The patient is a 37-year-old male who had skin grafts and reconstructive surgery complications following necrotizing fasciitis of the perineum and the genitalia with 30% TBSA. He presented with complex wounds which closed in 4 months, contractures, hypertrophic scars, hypersensitivity, fecal incontinence, and sexual intimacy dysfunction. Treatment included pelvic floor approach, education, desensitization, vesical and bowel movement rehabilitation, adapted pressure garment, gel application, scar massage, cutaneous and myofascial stretching and manual therapy. There was an improvement in BSFAQ score (5 points), pelvic floor muscle tension, PT observed scar, resolution of fecal incontinence, reduced penile deviation in the frontal plane, decreased hypersensitivity and ability to engage in pain-free sexual intercourse after 2 months of treatment. The patient is still progressively accepting his new body image but feels more confident with sexual intimacy. The results for POSAS and sensitivity are still ongoing.

**Conclusions:**

The patient has improved scars as well as reduced penile deviation, and sensitivity allowing masturbation and sexual intercourse without pain, which helped him regain intimacy with his partner.

**Applicability of Research to Practice:**

This is the first report of a standardized evaluation and treatment approach for the rehabilitation of skin graft complications after necrotizing fasciitis involving the perineum, genitalia and/or buttocks. Rehabilitation interventions improved his sexual function, body image satisfaction, and quality of life.

